# The Small Intestinal Microbiota and the Gut–Brain Axis in Parkinson’s Disease: A Narrative Review

**DOI:** 10.3390/biomedicines13071769

**Published:** 2025-07-19

**Authors:** Gloria Carrossa, Valentina Misenti, Sofia Faggin, Maria Cecilia Giron, Angelo Antonini

**Affiliations:** 1 Department of Pharmaceutical and Pharmacological Sciences, University of Padova, 35131 Padova, Italy; sofia.faggin@unipd.it (S.F.); cecilia.giron@unipd.it (M.C.G.); 2 Neurodegenerative Disease Unit, Centre for Rare Neurological Diseases (ERN-RND), Department of Neuroscience, Padua Neuroscience Center (PNC), University of Padova, Via Giustiniani, 5, 35128 Padova, Italy; valentina.misenti@unipd.it; 3 IRCCS, San Camillo Hospital, Via Alberoni, 70, Lido, 30126 Venice, Italy

**Keywords:** microbiota, Parkinson’s disease, probiotics, microbiota–gut–brain axis

## Abstract

Researchers are increasingly focusing on understanding the microbiota’s influence on disease susceptibility and overall health. The vast number of microorganisms in our gastrointestinal tract and their extensive surface area underscore their undeniable impact on well-being. Viewing the gut microbiome as a distinct pool of microbial genetic information that interacts with the human genome highlights its pivotal role in genetically predisposed diseases. Investigating this complex crosstalk may lead to the development of novel therapeutic strategies—such as targeting dysbiosis—to complement conventional treatments and improve patient care. Parkinson’s disease (PD) is a multifactorial condition originating from a combination of genetic and environmental risk factors. Compelling evidence points to the enteric nervous system as an initial site of pathological processes that later extend to the brain—a pattern known as the ‘body-first’ model. Furthermore, most patients with PD exhibit both qualitative and quantitative alterations in the composition of the gut microbiota, including dysbiosis and small intestinal overgrowth. Nonetheless, the existing literature predominantly addresses fecal microbiota, while knowledge of upper intestinal sections, like the duodenum, remains scarce. Given the potential for microbiota modulation to impact both motor and gastrointestinal symptoms, further research exploring the therapeutic roles of balanced diets, probiotics, and fecal transplants in PD is warranted.

## 1. Introduction

The global population is aging, but the factors contributing to normal versus pathological processes are still uncertain. Finding a comprehensive definition that fully explains the concept of aging is difficult, considering that this is not a single reductionist phenomenon based on a unidirectional pathway, as many try to describe [1].

In recent years, the concept of inflammaging refers to a low-grade systemic inflammation in the elderly that often persists without clear clinical symptoms [2,3]. This is interesting considering that many diseases typical of the elderly share an inflammatory pathogenesis and an asymptomatic stage.

Furthermore, age is the primary risk factor for neurodegenerative diseases such as Parkinson’s and Alzheimer’s, as evidenced by their rising prevalence in the elderly. Among the many alterations that occur with aging, one concerns the microbiota [4]. An altered balance between beneficial and pro-inflammatory bacteria has been observed in aged mice and is believed to be associated with degeneration of the enteric nervous system (ENS) during aging [5], suggesting an interaction between commensal microorganisms and neurodegenerative diseases.

Dysbiosis may not only arise as part of the physiological aging process, but it may also result from inflammaging itself, representing an adaptation of the microbiota to the changes induced by this chronic inflammatory state. Adding an additional layer of complexity, there is growing evidence that the pathological condition is associated with changes in the gut microbiota due to lifestyle-related variations. Discriminating the primary cause of dysbiosis among the various hypotheses proposed in affected patients remains a significant challenge for research.

Several studies have demonstrated the effectiveness of microbiota-targeted interventions for various neurological disorders. For instance, treating dysbiosis in patients with multiple sclerosis can reduce inflammation and reactivate the immune system [6]. Similar research is also promising in Alzheimer’s disease [7,8].

Considering these findings and the fact that, in recent years, the prevalence of Parkinson’s disease (PD) has been increasing more rapidly than other neurodegenerative disorders, a phenomenon referred to as the ‘Parkinson’s pandemic’ [9,10], it is crucial to acknowledge potential contributing factors. These include an aging population [9], environmental exposures [11], and lifestyle changes [12], such as dietary and smoking habits. In this context, exploring microbiota-targeted therapies is becoming increasingly relevant in PD research.

This review systematically analyzes the interactions between the gut–microbiota–brain axis and PD, exploring mechanisms influencing disease onset and progression, with particular emphasis on the small intestine. Furthermore, we present an updated overview of current scientific knowledge on potential therapeutic strategies to modulate this axis, highlighting its potential clinical implications and future perspectives in PD treatment.

Although numerous narrative and systematic reviews have examined the role of gut microbiota in PD [13,14], they have primarily focused on the overall gut microbiota, without specifically considering the role of the small intestine. Conversely, emerging evidence highlights the critical importance of the small intestine in PD pathogenesis, supporting the ‘body-first’ hypothesis that gut dysfunction may precede and contribute to neurodegeneration. Furthermore, the small intestine plays a pivotal role in the absorption and metabolism of PD medications, processes that are significantly influenced by microbial activity in this region. This review aims to address these gaps by focusing on the unique contribution of the small intestine to PD progression and treatment efficacy, offering novel insights into potential therapeutic targets and interventions.

## 2. From Composition to Clinical Relevance: Understanding the Gut Microbiota

The gut microbiota is the collection of bacteria, archaea, fungi, and viruses that inhabit our gastrointestinal tract [4]. It is estimated that each person has about 3.8 × 10^13^ bacterial cells throughout the body, equivalent to the number of human cells, thus meaning that each of us has a ratio of bacteria to human cells closer to 1:1 [15].

In the microbiota of a healthy individual, there are mainly strict anaerobes, and up to 50 different bacterial phyla can be identified, although *Bacteroidetes* and *Firmicutes* are most dominant [16]. The functions performed by these microorganisms vary, ranging from their contribution to metabolism to protection against pathogens. Recently, research has focused on the discovery of the role of the gut microbiota in maintaining health as well as in favoring the development of several diseases, including neurodegenerative conditions [16].

The gut microbiome, on the other hand, is the collective genetic information contained within the microbiota [17]. The number of genes encoded by the bacteria residing in the gut is approximately one hundred times that of the host individual, with 3.3 million genes identified compared to the 22,000 genes comprising the entire human genome [18]. These data are even more interesting if interpreted from the viewpoint of interindividual diversity: while each person shares 99.9% of their genetic heritage with others, they differ by 80–90% in terms of the microbiome [18]. Therefore, adopting a different perspective where we consider the human genetic heritage as the sum of human and microbial traits, we understand the importance of characterizing and deepening aspects related to the microbiome, which is one of the main objectives of the Human Microbiome Project (HMP) [19].

Microbial composition analyses commonly utilize the 16S rRNA marker gene. This choice stems from its ubiquitous presence across microorganisms and its optimal balance between a conserved sequence, enabling accurate alignment, and sufficient variation for robust phylogenetic analysis [19]. The information derived from this type of analysis provides a valuable starting point but remains inherently limited. As in other areas of medicine, leveraging omics approaches offers a more comprehensive perspective of the microbiota, not as a collection of individual components but as a complex ecosystem, where interactions are examined not only among microorganisms but also between them and the host. Currently, studies utilizing these technologies are still limited [20,21]; however, as their number increases, they will provide more detailed insights into metabolic pathways and bioactive compounds, contributing to a deeper understanding of the microbiota’s role.

Given the gut microbiome’s complexity, a deeper understanding requires further substantial research. However, continued advancements in this direction could leverage its immense diversity to develop personalized, targeted therapies for individual patients [18]. Numerous studies have documented dysbiosis across various diseases, including neurodegenerative disorders such as PD, further emphasizing the gut microbiota’s pivotal role in health. Notably, recent high-quality investigations, encompassing meta-analyses and large-cohort studies, consistently report significant alterations in the gut microbiota composition of PD patients [22,23,24,25]. These studies consistently document an increase in opportunistic genera, such as *Akkermansia*, and a reduction in short-chain fatty acid (SCFA)-producing bacteria, including *Lachnospiraceae* and *Faecalibacterium*. This microbial profile suggests a pro-inflammatory state, with a rise in mucin-degrading taxa and a loss of beneficial strains. These changes further support the hypothesis that intestinal dysbiosis may contribute to PD pathogenesis through mechanisms linked to gut inflammation.

While geographical and individual microbiota variations explain some differences between studies, disease-related alterations in PD remain significant after adjusting for confounders like age, diet, body mass index (BMI), constipation, and medication. This dysbiotic profile offers new insights for understanding and managing PD.

### 2.1. Segmental Dysbiosis in the Small Intestine of PD Patients

Current knowledge on small intestinal dysbiosis in PD remains limited. However, growing evidence highlights the importance of this region, suggesting that small intestine dysbiosis may directly affect both the pharmacokinetics of PD medications and the disease’s etiology and progression. In particular, up to 54% of PD patients show small intestinal bacterial overgrowth (SIBO), which is associated not only with gastrointestinal symptoms but also with more severe motor fluctuations [26]. Furthermore, recent preclinical evidence supports the body-first model, showing that fecal transplants from PD patients into mice can induce small intestinal dysbiosis, local inflammation, reduced Th17 cell populations, increased intestinal permeability, and ultimately, neuroinflammation and α-synuclein accumulation [27]. These findings suggest that the small intestine microbiota may represent one of the earliest points of initiation in the disease cascade, a possibility increasingly supported by clinical studies. In one investigation, duodenal fluid was collected via nasoduodenal tube from PD patients and controls, both fasting and postprandially. Although the luminal environment appeared broadly similar, signs of dysbiosis—such as elevated microbial metabolites—were observed, potentially affecting digestion and drug absorption [28]. Another study analyzing duodenal mucosal biopsies observed increased levels of pro-inflammatory *Proteobacteria* (e.g., Ralstonia) in PD patients, along with reduced beneficial taxa. Notably, the accumulation of oligomeric α-synuclein in the mucosa correlated positively with the abundance of Ralstonia, supporting the idea that local dysbiosis may contribute to inflammation and early α-synuclein pathology within the ENS. Overall, these data suggest that PD-related dysbiosis and inflammation may be segmental, with the small intestine playing a key role in disease onset [29]. Finally, a non-invasive tool such as the SIMBA capsule—currently being employed in an observational clinical study in PD patients (trial NCT06003608)—offer a promising new approach to directly sample the small intestinal microbiota in vivo. Table 1 summarizes the main studies mentioned above that have investigated the impact of the small bowel microbiota in PD to date.

### 2.2. The Effects of Drugs on the Microbiota

Growing research into drug–microbiota interactions has led to increased awareness of their complex relationship. A bidirectional communication between these entities is now widely recognized. Firstly, pharmacological agents can indirectly influence microbiota composition and activity by altering local microenvironments. A notable example is the administration of proton pump inhibitors (PPIs), which, by increasing gastric pH, facilitate the abnormal translocation of oral microbiota to the intestine, thereby inducing dysbiosis through the disruption of established commensal gastrointestinal microbial distribution [30]. Another mechanism by which drugs may alter the intestinal microflora involves promoting the growth of specific bacterial species or, conversely, reducing their numbers—an effect observed even with non-antibiotic drugs that exhibit antimicrobial activity [31]. On the other hand, microorganisms can also influence drugs, giving rise to the concept of pharmacomicrobiomics [32,33]. The gut microbiota can modify both the pharmacokinetics and pharmacodynamics of a drug, potentially altering its efficacy and safety profile, leading to side effects or even adverse reactions. This is achieved either through direct drug transformation or by modulating metabolism and/or the immune system [34,35]. Indeed, gut microorganisms can produce enzymes involved in drug biotransformation reactions or even generate molecules that compete with the drug for the same substrates [36]. A study identified 70 drug–microbiota interactions, highlighting that many of them are related to the phenomenon of bioaccumulation. This process has been observed across a wide range of drugs, including antidepressants, antidiabetics, and cardiovascular medications. Bioaccumulation can alter pharmacokinetics and reduce therapeutic efficacy by affecting the drug bioavailability. Additionally, this phenomenon may change the composition of the microbiota, promoting the growth of bacteria that could modify the body’s response to drugs, supporting the hypothesis that personalized therapeutic approaches are essential for optimizing treatments [37].

**Figure 1:**
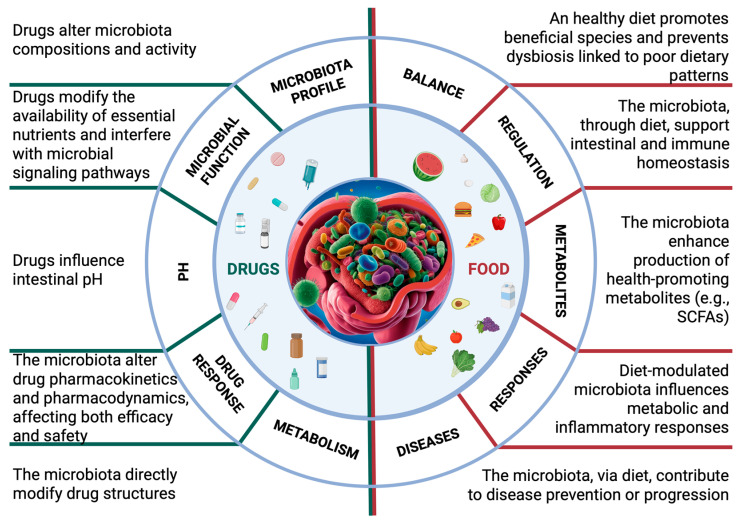
Schematic representation of key microbial changes and their corresponding impacts on the efficacy of diet and drugs. Alterations in microbiota composition, activity, and function can disrupt microbial balance and influence drug action, nutrient digestion, and overall patient outcomes. These changes may affect gut permeability, systemic inflammation, and the absorption and metabolism of nutrients and drugs, potentially contributing to side effects or disease onset. (Image created with ©BioRender.com and ©Canva 2025).

It is intuitive to assume that antibiotics, which directly target bacterial cells, can significantly alter the gut microbiota. The overuse of antibiotics has been observed to cause the development of many disorders associated with intestinal dysbiosis [38]. Since most commercially available antibiotics have broad-spectrum activity, their effects are not limited to pathogens but also impact the healthy gut flora [39]. Consequently, resistant bacteria may develop, further disrupting the microbiota balance [40]. Less obvious is the idea that even non-antibiotic drugs can lead to similar alterations. However, numerous studies have already demonstrated this association [41,42]. Given the rising number of patients undergoing polypharmacotherapy, a recent study investigated the effects of multi-drug therapy and provided evidence of widespread changes in metabolic potential, taxonomy, and resistome associated with commonly used medications, further reinforcing previous findings [43]. However, as shown in Figure 1, pharmacological treatments are only one of the many factors that can impact the gut microbiota.

### 2.3. The Impact of Diet on the Microbiota

Due to its considerable interindividual variability, the gut microbiota can be regarded as a unique biological marker. Given the great diversity within this ecosystem, there is no single configuration that can be defined as a “healthy microbiota” [17]. This suggests that various approaches can benefit gut health. Well-balanced diets like the Mediterranean, high-fiber, or balanced plant-based diets significantly modify microbial composition and are potentially linked to enhanced well-being [44]. Conversely, an unbalanced diet causes various types of dysbiosis. Since the gut microbiota’s role in health is now widely recognized, poor dietary habits clearly contribute to a broad range of disorders. Alpha diversity appears to be a reliable indicator of gut bacterial ecosystem health [45]. This measure increases significantly until adulthood, and many diverse diseases share reduced alpha diversity as a common feature [46]. Higher consumption of refined sugars, processed foods, and other Western diet components correlates with decreased gut microbiota diversity [47]. Conversely, adopting the Mediterranean diet as a lifestyle enhances both microbial diversity and richness [48]. Many factors influencing the gut microbiota are established early in life, including the mode of delivery [49] and maternal or early childhood diet [50]. For example, the gut microbiota of children with a normal or high BMI tends to show greater diversity compared to that of underweight children [51]. In contrast, in adults, the pattern appears reversed—overweight or obese individuals, or those with a high BMI, often exhibit reduced alpha diversity [52,53]. These observations highlight that, in order to effectively modulate the gut microbiota to support overall health, it is more beneficial to focus on long-term dietary patterns rather than isolated nutrient interventions, which may be promising but still require further investigation.

## 3. The Microbiota–Gut–Brain Axis: Foundations and Physiological Implications

Over the past 70 years, numerous studies have examined the interactions between two complex systems—the gut and the brain—introducing and gradually reinforcing the concept of the “gut–brain axis” [54,55,56]. These early findings have been further supported by physiological experiments, advanced experimental techniques [57], and investigations using functional magnetic resonance imaging (fMRI) [58]. Together, this body of research has revealed a close interconnection between the central nervous system (CNS) and the ENS. More recently, growing interest in the role of gut microorganisms has led to a broader perspective, culminating in the concept of the “microbiota–gut–brain axis” [59], to highlight the bidirectional communication between these three components.

Microorganisms can influence gut barrier, motility, and secretion, which, in turn, affect brain function. Conversely, the brain can modulate the gut environment and microbiota composition through neural, endocrine, and immune pathways [59].

These new findings allow us to identify various therapeutic applications of the microbiota–gut–brain axis, such as the use of neuromodulators in the treatment of digestive disorders, both to manage pain and address the inflammatory component [60]. Some early observations also suggest the possibility of treating brain disorders with microorganisms. For example, fecal transplantation has been shown to be effective in relieving the symptoms of autistic patients with digestive problems and dysbiosis, leading to a decrease in both neurological and gastrointestinal symptoms [61].

An innovative approach is the use of optogenetic technology. Originally developed to investigate the gut–brain interconnections, it has also been found to enable precise control over gut microbiota metabolism and the regulation of genetically engineered bacteria for therapeutic purposes [62]. Therefore, the microbiota–gut–brain axis represents a promising therapeutic target for a variety of pathological conditions, including neurological diseases. However, further research is essential to deepen our understanding, enhance the reliability of findings, and enable their translation into routine clinical practice.

## 4. Involvement of the Microbiota–Gut–Brain Axis in Parkinson’s Disease

PD is named after the British physician James Parkinson, who first described its key features in his 1817 work, *An Essay on the Shaking Palsy*. PD is a progressive neurodegenerative disorder and one of the most disabling conditions affecting the CNS [63,64]. The pathological hallmark of PD is the deposition of aggregated α-synuclein in the neurons, so-called Lewy bodies [65], and progressive loss of striatal dopamine nerve terminals resulting in dopamine depletion [65,66]. Manifestations of PD include motor symptoms and non-motor symptoms (NMS). The signs that most characterize the pathology are bradykinesia, resting tremor, rigidity, and postural instability. In addition to these, patients are subjected to secondary motor dysfunction such as gait impairments, micrographia, speech difficulties, dysphagia, and dystonia [66]. It has been observed that certain enteric clinical manifestations, leading to bloating, constipation, nausea, or weight loss, occur in PD patients many years before the appearance of motor symptoms [67]. There are several risk factors that predispose individuals to the onset of PD, many of which share the ability to influence the gut microbiota, suggesting a possible interaction between them [68]. For the initial evaluation of the involvement of the gut–brain axis in PD, the contribution of preclinical research has been fundamental. It has been shown that germ-free mice exhibit dysregulated dopamine activity in various areas of the brain [69]. Indeed, the gut microbiota can produce various neurotransmitters, including dopamine [59]. Alterations in the gut microbiota may negatively affect the immune response, thereby influencing neuroinflammation. Under conditions of dysbiosis, systemic inflammation can occur, potentially triggering protein aggregation that may propagate to the brain via the vagus nerve—the so-called microbiota–gut–brain axis through which the microbiota influences brain activity and function [70].

In PD, accumulations of phosphorylated α-synuclein are initially found in the ENS and may reach the CNS through the vagus nerve, which itself does not appear to suffer direct damage [71,72]. These observations suggest that the ENS facilitates the spread of the disease [72]. However, further studies are needed to definitively determine whether this represents a key pathogenetic event in PD. Based on current evidence, it is believed that such interactions contribute to disease development, albeit with interindividual variability.

If the enteric accumulation of pathological α-synuclein is replicated in experimental models, it subsequently appears in the brain; conversely, if α-synuclein pathology originates elsewhere, it still spreads to the ENS, causing damage there [73]. In light of these observations, it appears plausible that the bidirectional interaction between the gut and the CNS in the pathogenesis of PD is significantly influenced by intestinal dysbiosis, which leads to altered microbial metabolic activity, further supporting the hypothesis that modulating the gut–microbiota–brain axis may contribute to improving the condition of PD patients.

NMS are common in PD and negatively impact patient quality of life, often requiring dedicated management. In PD patients, dysbiosis is notably marked by a reduction in butyrate-producing bacteria and an elevation in *Collinsella.* This microbial pattern has been documented in both PD patients and their first-degree relatives exhibiting REM sleep behavior disorder (RBD) [74]. Moreover, dietary modifications, including increased fiber and reduced sugar consumption, can alter the gut microbiota and improve NMS [75]. These observations imply that gut dysbiosis might precede the manifestation of motor symptoms, potentially serving as an early biomarker for PD.

### 4.1. The Small Intestine in Parkinson’s Disease: A Crucial Site for Pathogenesis

The previously mentioned dysbiosis in the small intestine of PD patients (see Section 2.1) is only one of the recent scientific findings that identify the small intestine as a critical site in the pathogenesis of PD. An interesting aspect is the distribution of α-synuclein across various segments: the rostrocaudal gradient of α-synuclein pathology shows an 83.6% frequency in the upper gastrointestinal tract compared to 64.3% in the lower gastrointestinal tract [76]. Alterations have also been observed in the ENS at the duodenal level: immunohistochemical analysis revealed reactive gliosis with a significant increase in glial cell density and cell size in the duodenum of patients with advanced PD compared to healthy controls. Enteric glial cells (EGCs) showed co-localization of GFAP and S100β, while SOX10 immunostaining revealed a sparse and sporadic distribution. The correlations with α-synuclein suggest that intestinal inflammation, mediated by EGCs, is associated with synucleinopathy in advanced PD [77]. Moreover, chronic intestinal inflammation and increased gut permeability (“leaky gut”) are strongly related to neurodegenerative processes in PD. The observed reactive gliosis, misfolded α-synuclein in the ENS, and the presence of clusters of T and B lymphocytes in duodenal biopsies from PD patients further support the hypothesis that enteric inflammation may precede and promote α-synuclein pathology via the gut–brain axis [78]. Furthermore, it has been demonstrated that multiple biopsies from different duodenal segments improve diagnostic yield [79]. A recent study found that damage to the upper gastrointestinal mucosa increases the risk of PD by 76% in a cohort of 9350 patients [80]. Despite this, there is a gap in the literature regarding studies directly comparing functional differences between the duodenum, jejunum, and ileum in PD patients. This highlights a critical unresolved question that requires focused investigation. Considering that the small intestine plays a crucial role in PD pathogenesis, the local alterations observed in this region are not only crucial for understanding disease progression but may also influence treatment responses. A deeper understanding of these mechanisms is essential for the development of targeted diagnostic and therapeutic strategies.

### 4.2. Gut Microbial Products Influence α-Synuclein Aggregation and Neurotoxicity

The accumulation of fibrillar α-synuclein is a key pathological hallmark of PD. These insoluble amyloid fibrils originate from oligomeric intermediates that are highly prone to aggregate and propagate from neuron to neuron through conformational templating. In addition to disrupting membrane integrity, α-synuclein aggregates are directly neurotoxic and play a central role in disease progression. Oligomeric and protofibrillar α-synuclein species, which are rich in β-sheet structures, are considered the most neurotoxic conformers, being more harmful than mature fibrils. These assemblies damage neuronal membranes through pore formation, trigger oxidative stress and inflammation, and impair mitochondrial and synaptic functions. Importantly, α-synuclein aggregates can spread between cells and act as templates for further aggregation, amplifying pathology across neural circuits [81].

Recent evidence suggests that amyloid components from bacterial biofilms may interact with α-synuclein. These interactions could modulate its aggregation, affect its prion-like behavior, and increase its neurotoxic potential. The structural similarity among amyloid fibrils suggests that shared aggregation mechanisms may allow for interactions between different amyloidogenic proteins. In light of this, one study investigated whether amyloidogenic proteins from gut bacteria could accelerate α-synuclein aggregation and worsen motor function and neuroinflammation in mouse models of PD. The results showed that curli, produced by *E. coli*, increases α-synuclein aggregation in both the gut and brain. This leads to more severe motor deficits and gastrointestinal dysfunction in Thy1-αSyn transgenic mice. These effects were not observed with a mutant *E. coli* strain that cannot produce curli. This mechanism seems to involve the ability of bacterial amyloid proteins, such as curli, to act as cross-seeding agents. They promote α-synuclein aggregation through prion-like processes. This contributes to neuroinflammation, intestinal dysfunction, and motor impairment. It suggests that specific microbial components may worsen α-synuclein-related pathology and its associated symptoms [82]. These initial findings have been confirmed by additional studies [83]. Following Sampson et al., other researchers found that Pseudomonas produces amyloid proteins like FapC in its biofilms. It was also shown that if an amyloid protein aggregates faster than α-synuclein, it can act as a seed and promote its fibrillation. Conversely, slower aggregation kinetics may have an inhibitory effect [84].

Further in vivo studies are needed to confirm these effects, identify biologically relevant concentrations, and evaluate whether blocking or removing these molecules could prevent the onset of α-synucleine-related pathology.

## 5. The Role of Gut Microbiota in the Pharmacological Management of Parkinson’s Disease

In light of the previously discussed overview of the main pathogenic mechanisms underlying PD, the rationale behind the three principal therapeutic strategies currently employed in its management becomes more evident. These include oral pharmacological treatments based on L-DOPA, dopamine agonists, and monoamine oxidase type B inhibitors (MAO-BIs). Additionally, catechol-O-methyltransferase (COMT) inhibitors are commonly used in clinical practice in combination with L-DOPA, aiming to reduce its peripheral metabolism, thereby prolonging its therapeutic efficacy.

Although all of these options are considered valid first-line strategies, L-DOPA is associated with superior therapeutic efficacy in clinical practice [85] and is, therefore, generally preferred over alternative treatments. This predominant use may account for the relatively greater number of studies investigating the interactions between L-DOPA—a dopamine precursor—and the gut microbiota. By contrast, as highlighted in our analysis, specific experimental evidence exploring the interactions between the gut microbiota and dopamine agonists or MAO-BIs remains limited to date.

### 5.1. Levodopa

One of the most evident pathogenic alterations in PD is the progressive loss of dopaminergic neurons, which produce the neurotransmitter dopamine, in the brain substantia nigra pars compacta (SNc), leading to reduced dopamine concentrations in the striatum [65]. Consequently, a rational therapeutic strategy involves the restoration of appropriate levels of dopamine. However, due to its chemical structure, dopamine is unable to cross the blood–brain barrier, necessitating the use of its precursor, L-DOPA, which remains the gold standard in PD treatment. Despite its clinical efficacy, orally administered L-DOPA presents significant limitations in terms of bioavailability. Owing to extensive first-pass metabolism in the small intestine, particularly in the duodenum and proximal jejunum—its primary site of absorption—and subsequent peripheral conversion, only approximately 1–5% of the administered dose effectively reaches the CNS [86]. In addition to reduced therapeutic efficacy, peripheral metabolism of L-DOPA results in the production of metabolites that contribute to adverse effects [87]. For this reason, simply increasing the dosage is not a viable strategy for overcoming its limited bioavailability.

Interestingly, these biotransformations are mediated by enzymes that, besides being expressed in enteric mucosa, may be encoded by specific bacterial species within the gut microbiota. For instance, some studies have demonstrated that *Enterococcus faecalis* expresses tyrosine decarboxylase (TDC), an enzyme capable of converting L-DOPA into dopamine [88,89]. Additionally, the same researchers observed similar activity in *Enterococcus faecium* [88]. These observations suggest that a higher abundance of gut bacteria expressing TDC in the small intestine may impair the absorption of the L-DOPA/carbidopa combination. This implies the existence of interindividual variability in drug efficacy, potentially attributable to differences in gut microbiota composition. Indeed, the study by Van Kessel et al. (2019) reported a positive correlation between the relative abundance of the bacterial TDC gene and both the daily L-DOPA dose and disease duration [89]. Supporting this finding, a subsequent study involving PD patients showed that moderate responders to L-DOPA exhibited a higher abundance of the TDC gene and *Enterococcus faecalis* compared to good responders [90]. However, these and similar studies share a significant methodological limitation: the quantification of the TDC gene was performed on fecal samples. It is well established that L-DOPA absorption primarily occurs in the proximal small intestine, where synuclein pathology involves the ENS [91], and that gut microbiota composition varies markedly along the gastrointestinal tract. Consequently, analyses based solely on fecal samples may not accurately reflect microbial activity at the site of drug absorption, thus limiting the validity of the conclusions drawn from these studies.

Given the evidence that L-DOPA is inactivated by decarboxylase activity, current commercial formulations co-administer this dopamine precursor with inhibitors such as carbidopa, benserazide, or methyldopa. These compounds are intended to inhibit peripheral decarboxylation and enhance central availability. However, none of these inhibitors has demonstrated a sufficiently effective inhibitory action against the bacterial TDC enzyme [89].

**Figure 2:**
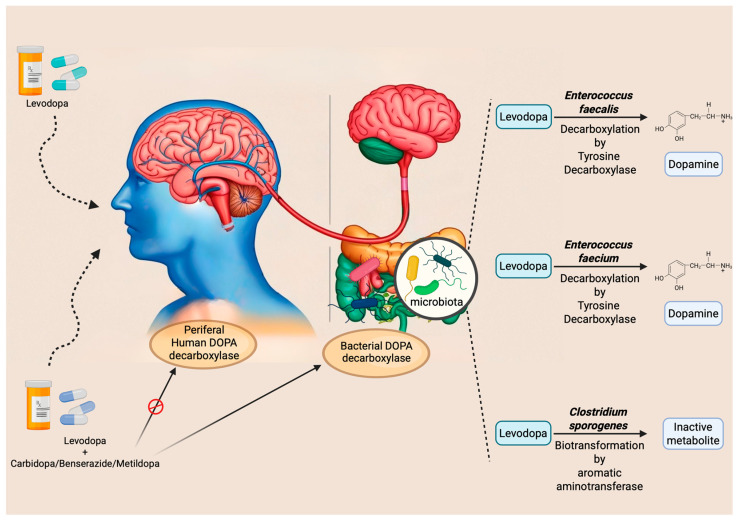
This graphical overview illustrates the interaction between L-DOPA and the gut microbiota, highlighting the main microbial species involved in the drug’s metabolism. It also distinguishes between L-DOPA monotherapy and co-administration with decarboxylase inhibitors, showing how the latter can reduce premature conversion of L-DOPA into dopamine, thereby potentially improving its systemic bioavailability. However, the efficacy of peripheral decarboxylase inhibitors exhibits significant interindividual variability, likely due to their inability to inhibit the bacterial enzyme responsible for L-DOPA metabolism. As a result, higher doses may be required in some patients to achieve an adequate therapeutic effect. This illustration underscores the role of the gut microbiota in L-DOPA pharmacokinetics and highlights the importance of considering microbe–drug interactions in PD management (Image created with ©BioRender.com and ©Canva 2025).

Beyond modulating absorption profiles, the gut microbiota also plays a significant role in the interindividual variability of side effects manifestation. For example, *Clostridium sporogenes* has been shown to mediate a specific biotransformation of L-DOPA by producing aromatic aminotransferase. This enzyme utilizes unabsorbed intestinal L-DOPA as a substrate, leading to the formation of an inactive deaminated metabolite, which has also been implicated in the onset of gastrointestinal side effects [92]. These main mechanisms and interactions are summarized in Figure 2.

Identifying potential targets of microbiota-mediated alterations in small intestine L-DOPA first-pass metabolism may contribute to the optimization of PD therapy by enhancing L-DOPA bioavailability and, consequently, improving its therapeutic efficacy while minimizing adverse effects. At present, the broader adoption of subcutaneous L-DOPA delivery systems offers a promising strategy to circumvent intestinal metabolic interference [93,94,95]. Such approaches may exert beneficial effects not only on gastrointestinal disturbances but also on the management of motor symptoms. Duodenal administration of L-DOPA (Duodopa^®^) is a well-established therapy for patients with advanced PD who are unable to control motor fluctuations with oral L-DOPA. Long-term reduction in motor fluctuations and dyskinesias, leading to an improvement in the patients’ quality of life, is well documented, even if this may not modify disease progression and death, supporting a multifactorial etiology in PD [96]. From a pharmacokinetic perspective, duodenal drug administration allows for faster absorption compared to oral drug formulation, with reduced variability in plasma concentrations due to direct absorption in the small intestine, which has been identified as the primary site of absorption [97]. Considering the potential role of the small intestine in the pathogenesis of PD, it can be hypothesized that this mode of administration may further optimize therapeutic efficacy.

### 5.2. Dopamine Agonists

Although current evidence on the interactions between other classes of drugs used in PD treatment and the gut microbiota remains limited, further investigation in this area is warranted. Drug–microbiota interactions may significantly influence individual responses to therapy, potentially leading to variability in clinical outcomes.

Preclinical studies in animal models have suggested that treatment with dopamine agonists may contribute to reduced intestinal motility, potentially promoting the development of SIBO. According to van Kessel et al. (2022), these alterations were associated with an increased abundance of bacterial genera such as *Lactobacillus* and *Bifidobacterium*, alongside a reduction in species belonging to the *Lachnospiraceae* and *Prevotellaceae* families [98].

It is worth noting that in the aforementioned study, dopamine agonists were administered in combination with L-DOPA–carbidopa. Consequently, disentangling the specific effects of each pharmacological agent on gut microbiota composition and gastrointestinal motility remains an open question and a critical area for future research.

### 5.3. COMT Inhibitors

COMT inhibitors are used in the treatment of PD to extend peripheral L-DOPA bioavailability. Several studies have reported dysbiosis in patients undergoing treatment with COMT inhibitors, including an increased abundance of *Enterobacteriaceae* [99] and *Lactobacilluslacteae* [100], along with a decrease in *Bifidobacteria* [101] and *Lachnospiraceae* [100]. Collectively, these alterations reflect a microbial imbalance marked by an overrepresentation of potentially pathogenic species and a concomitant depletion of commensal bacteria with anti-inflammatory properties. This dysbiotic profile may play a key role in the onset of the gastrointestinal side effects commonly associated with COMT inhibitor therapy.

Notably, the use of entacapone has been found to be inversely associated with fecal levels of butyrate—one of the most abundant SCFAs produced by the gut microbiota [102]. Given the central role of SCFAs in modulating host physiological functions, including immune regulation and intestinal barrier integrity, further investigation into the implications of entacapone on SCFA metabolism is, therefore, of considerable interest.

## 6. Potential Strategies of Microbial Intervention in Parkinson’s Disease

### 6.1. Food (Diet, Prebiotics)

Making small dietary adjustments is relatively simple and accessible, and may also lead to relatively rapid changes in gut microbiota composition, sometimes observable within 24–48 h [103]. It has been observed that the rate at which these changes occur also depends on the stage of the disease. Specifically, in the early stages of PD, dietary habits can significantly influence the composition of bacteria, suggesting that during this phase, the diet may still modify the microbiota. In contrast, in the more advanced stages of the disease, changes in the microbiota may become more stable and less responsive to dietary interventions [75].

Evidence indicates that adhering to a healthy dietary pattern in PD correlates with reduced circulating lipopolysaccharide (LPS) levels—pro-inflammatory endotoxins typically elevated in affected patients and implicated in neurodegenerative processes [75,104]. These dietary habits can also augment SCFA-producing species, benefiting the intestine by strengthening the epithelial barrier and the CNS by mitigating neuroinflammation [75]. To achieve these effects and support the diet’s classification as a health-promoting PD intervention, adequate dietary fiber intake is essential, readily attainable through regular consumption of fiber-rich foods like vegetables, fruits, legumes, and whole grains.

A strategy consistent with these findings and proven beneficial for alleviating PD symptoms is the use of prebiotics. These compounds selectively promote beneficial host microorganism growth and activity, for instance, via direct sodium butyrate administration [105]. A recent clinical study investigated a four-week high-fiber diet supplemented with the prebiotic lactulose in PD individuals. The intervention resulted in a notable increase in Bifidobacteria, linked to a significant rise in fecal SCFA production and improvements in gastrointestinal symptoms, particularly constipation. Additionally, that study reported elevated neuroprotective metabolites, including S-adenosylmethionine, suggesting benefits beyond the gastrointestinal tract [106].

However, prebiotic research in PD treatment progresses much slower than probiotic research, remaining limited despite promising findings on prebiotic–probiotic associations (symbiotics), as discussed subsequently. Among bioactive compounds with potential therapeutic relevance in PD through gut microbiota modulation, polyphenols are particularly interesting. These molecules, characterized by potent antioxidant properties, are abundant in plant-based foods such as fruits, vegetables, tea, cocoa, extra virgin olive oil, and various spices, and are well-known for their neuroprotective effects.

Experimental studies have demonstrated that specific polyphenols—such as epigallocatechin gallate, the main catechin found in green tea, and curcumin—can inhibit α-synuclein aggregation and attenuate neuroinflammatory responses [107]. In addition to their direct antioxidant and anti-inflammatory actions, polyphenols also influence the gut microbiota by modulating its composition and metabolic activity. Notably, regular dietary intake of flavonoids has been associated with an increased production of SCFAs by intestinal microbes, further supporting their role in maintaining gut and brain health [108].

Dietary supplements also fall within this category. In particular, supplementation with omega-3 fatty acids (omega-3s) has been shown to exert beneficial effects on the CNS by supporting blood–brain barrier integrity, slowing the progression of neurodegeneration, and inhibiting neuroinflammatory processes. Moreover, omega-3s are believed to protect dopaminergic neurotransmission through mechanisms involving the inhibition of NF-κB signaling pathways [109,110]. The gut microbiota may also contribute to these effects by adopting a more anti-inflammatory profile in response to omega-3 supplementation; however, this hypothesis requires further validation through targeted research efforts [111]. A critical analysis of the current literature reveals a scientific gap between the extensive body of preclinical and observational studies and the limited availability of evidence-based dietary guidelines specifically aimed at modulating the development or progression of PD. Interventional studies in this area remain in the early stages and are still insufficient to support formal clinical recommendations.

### 6.2. Probiotics

Probiotics are defined by the World Health Organization as “the moderate intake of live microorganisms with beneficial effects on the host’s health” [112].

As scientific interest grew around microbiota modulation with probiotics in PD therapy, promising results emerged, especially for gastrointestinal symptoms like constipation, one of the most common non-motor features of the disease. The beneficial effects of probiotics are thought to arise from the introduction of specific bacterial strains or modulation of microbial abundance, which, in turn, leads to the production of metabolites capable of reinforcing the integrity of the intestinal mucosa [113] and inhibiting harmful bacteria [114]. Further evidence supporting the existence and functional relevance of the microbiota–gut–brain axis in PD comes from studies demonstrating that probiotics can exert effects not only at the gastrointestinal level but also within the CNS. In particular, certain probiotic strains have been shown to modulate neurotransmitter activity and exert neuroprotective effects on dopaminergic neurons [115]. Notably, a 2023 meta-analysis reported significant improvements in Unified Parkinson’s Disease Rating Scale (UPDRS) Part III scores, suggesting that probiotic supplementation may help reduce motor symptom severity and potentially influence the overall progression of the disease [116].

To explore the wide range of potential probiotic-based therapies in PD, distinguishing between single-strain and multi-strain probiotic formulations is essential. Several preclinical studies report promising outcomes with single-strain interventions. *Lactobacillus plantarum* DP189, administered for two weeks in an MPTP-induced murine model of PD, significantly reduced neuroinflammation and α-synuclein accumulation in the brain [117]. In the same model, oral administration of *Lacticaseibacillus rhamnosus* E9 produced both central and intestinal benefits, including increased cerebral dopamine levels, improved intestinal barrier integrity, and restoration of microbial balance [118]. *Bifidobacterium breve* efficacy has been demonstrated across two different strains in PD animal models. Strain CCFM1067 exerted neuroprotective effects by suppressing glial activation, simultaneously modulating the gut microbiota by reducing pathogenic bacteria like *Escherichia* and promoting beneficial genera such as *Akkermansia*, leading to increased SCFAs with anti-inflammatory properties [119]. Similarly, the *B. breve* Bif11 strain improved motor function and intestinal permeability [120]. A clinical study involving 82 PD patients evaluated the effects of the single-strain probiotic *B. lactis* Probio-M8, administered for 12 weeks alongside standard therapy. The results showed improvements in both motor and NMS (e.g., sleep quality, bowel regularity), accompanied by favorable changes in gut microbiota composition [121].

Regarding multi-strain formulations, a noteworthy clinical study investigated oral supplementation with a capsule containing *L. acidophilus*, *L. fermentum*, *L. reuteri*, and *B. bifidum* over three months. This intervention reduced the motor total score, suggesting clinical improvement. Moreover, a decrease in systemic inflammatory markers like high-sensitivity C-reactive protein was observed [122].

Despite these promising findings, probiotic use faces certain limitations. Most commercial probiotic products fail to reach the intestine due to inactivation by stomach acid. To address this issue, Symprove K-1803, a more advanced orally administered probiotic, has been developed to deliver live bacteria effectively to the intestinal tract [123].

Although some small studies suggest that probiotics may improve both motor and NMS, current evidence remains preliminary due to heterogeneous study designs, small sample sizes, and short follow-up periods. Some trials have failed to demonstrate sustained benefits or significant changes in the gut microbiota, indicating that probiotic-induced effects may be transient [124]. Therefore, more rigorous and long-term studies are essential to determine the true efficacy and safety of probiotics in PD.

### 6.3. Synbiotics

The combination of prebiotics and probiotics in a single formulation is referred to as a synbiotic. This dual approach appears to be more effective in supporting gut microbiota balance than the use of either component alone [125]. Current research primarily focuses on evaluating whether synbiotics administration can improve gastrointestinal symptoms associated with CNS disorders, including PD [126,127]. In a murine model of PD, an experimental synbiotic composed of polymannuronate and *Lactobacillus rhamnosus* GG demonstrated promising results following a five-week treatment regimen. The intervention preserved dopaminergic neurons and improved motor function, as evidenced by behavioral test outcomes. Notably, the synbiotic exerted greater neuroprotective effects than either component administered individually [128].

The positive outcomes of these studies encourage further exploration of the use of these therapies in PD.

### 6.4. Antibiotics

The primary goal of gut microbiota modulation is to restore a healthy balance between beneficial and harmful bacteria. One strategy within this approach focuses on targeting and reducing specific pathogenic taxa.

Since PD patients are often affected by SIBO [129], broad-spectrum antibiotics such as rifaximin and tetracyclines have proven effective in its eradication [130,131]. Importantly, SIBO may impair L-DOPA metabolism by promoting the overgrowth of gut bacteria expressing the TDC gene, thereby reducing L-DOPA bioavailability [132]. This suggests that eliminating SIBO may enhance L-DOPA absorption and, in turn, improve motor symptoms. However, a clinical study on rifaximin treatment for SIBO found no significant effects on L-DOPA pharmacokinetics [26].

Beyond their antimicrobial activity, certain antibiotics have also shown neuroprotective properties. In a preclinical study, Zhou and colleagues (2021) demonstrated that ceftriaxone exerted anti-inflammatory effects in a murine model of PD, highlighting its potential CNS benefits [133]. Clinically, a combination therapy involving a sodium phosphate enema followed by oral rifaximin and polyethylene glycol was associated with reduced motor fluctuations and a significant improvement in dyskinesia severity and duration in PD patients [134]. An interesting strategy, currently under evaluation (Trial ID: 2024-510629-24-00), is to reduce the gut bacteria that decarboxylate L-DOPA by administering antibiotics, such as rifaximin, for potentially increasing the bioavailability and effectiveness of L-DOPA in PD patients. While these findings are promising, it remains unclear whether the observed benefits stem from microbiota modulation or other mechanisms of action.

Should future studies confirm these effects, careful evaluation of the risk–benefit profile of long-term antibiotic use in this patient population will be essential before considering their implementation as a viable therapeutic strategy.

### 6.5. Amyloid Inhibitors

As previously reported in Section 4.1, microbial amyloids can influence α-synuclein aggregation and toxicity through cross-seeding mechanisms. These findings have increased interest in targeting bacterial amyloids and their biosynthetic pathways. Recent studies focused on pharmacological strategies to block their interaction with α-synuclein, aiming to reduce fibril formation, decrease neurotoxicity, and potentially slow disease progression. Curli fibers produced by *Escherichia coli* are among the most studied microbial amyloids as potential pharmacological targets. In vitro studies have shown that several periplasmic chaperones—including CsgC, CsgE, Spy, DnaK, and Hsp33—can inhibit amyloid formation of the CsgA subunit. Notably, CsgC can also block α-synuclein aggregation without affecting Aβ42 fibrillization [135].

In addition to protein-based inhibitors, small molecules like 2-pyridones have been developed to prevent amyloid assembly by diverting CsgA into off-pathway oligomers. More recently, the regulatory protein CsgI (also known as YccT) has been identified as a curli inhibitor, acting by both blocking CsgA polymerization and reducing curli gene expression [136]. In mouse models, curli inhibition has shown encouraging results. In Thy1-αSyn transgenic mice, oral treatment with a gut-restricted amyloid inhibitor reduced α-synuclein aggregation, neuroinflammation, and motor deficits [82].

Furthermore, the amyloid protein FapC from Pseudomonas, though less studied, shows similar therapeutic potential. In vitro models have demonstrated that the disordered chaperone FapA significantly slows FapC fibrillation [137], and the proteolytic enzyme serrapeptase has been found to reduce both biofilm formation and amyloidogenesis at micromolar concentrations [138].

Altogether, these findings suggest that microbial amyloids may serve as promising therapeutic targets. However, further in vivo studies are needed, especially on FapC, to better assess the long-term effects and clinical relevance.

## 7. Potential Developments

Although research is still preliminary, the microbiota is increasingly recognized as a potential PD biomarker [10]. Identifying reliable microbiota signatures could improve clinical diagnosis, even in the premotor phase, a period characterized by marked gastrointestinal symptoms like constipation and nausea [139]. Early intervention is crucial for ensuring effective, long-lasting treatments without waiting for the emergence of motor symptoms, which indicate advanced disease stages.

If validated, these hypotheses position the microbiota as a potential therapeutic target—via probiotics, prebiotics, or fecal transplantation—for symptom relief, disease progression modulation, or adjunctive treatment to reduce CNS drug side effects [72].

Several microbiota-modulating therapeutic strategies show promising potential in PD. However, to confirm their efficacy, further clinical studies involving larger patient cohorts and focused analysis of the small intestine microbiota’s influence are needed. It is evident that after initial exploratory research, the field must urgently move from observational studies to interventional clinical trials. This transition is essential to draw robust conclusions and better define the impact of these therapies.

Increasing attention is also being paid to the role of the gut microbiota and dietary proteins in the initiation and progression of α-synuclein pathology. Cross-reactivity between α-synuclein and microbial or dietary proteins via cross-seeding mechanisms may be a critical factor in the neurodegenerative cascade. Certain gut microbes, such as curli-producing *Enterobacteriaceae*, appear to act as permissive—not primary—factors, accelerating toxic α-synuclein aggregation in vulnerable individuals. In parallel, soluble microbial products like rhamnolipids and LPS may serve as molecular triggers with direct neurotoxic potential. Targeting highly pathogenic CsgA variants or specific microbial metabolites could represent a promising avenue for prevention or intervention. Further research is necessary to clarify molecular mechanisms, establish physiologically relevant concentrations, and evaluate whether modulation of diet or microbiota can prevent disease onset.

Ultimately, enhancing our understanding of the microbiome—specifically, the genetic information encoded within the microbiota of the small intestine—holds significant promise for advancing our knowledge of PD, where genetic factors play a central role in both predisposition and disease progression.

As scientific evidence continues to accumulate, it is conceivable that the research community will recognize a fundamental shift in perspectives regarding neurodegenerative diseases. The gut–brain axis is not merely an intriguing area of research; it is a pivotal element, especially in PD, where gastrointestinal alterations are not secondary manifestations but integral components of the pathological process, with profound implications for both disease progression and clinical management.

Despite this growing body of evidence, many studies—particularly those focusing on dysbiosis in PD—continue to rely primarily on fecal microbiota samples, which are now well-established as not reflecting the composition of the small intestine microbiota. This gap in research is critical, as evidenced by various studies demonstrating the crucial role of the small intestine in PD, e.g., the spreading of α-synuclein across various segments [76], the morpho-functional alterations of the ENS [77], and the main site of L-DOPA absorption. Moreover, conditions like SIBO, reported in a variable percentage of PD patients (up to 54%) [26], further highlight the need for studies that specifically address the small intestine microbiota. In conclusion, the synthesis of evidence presented in this narrative review has elucidated the pivotal role of the small intestine in PD. Consequently, future research should prioritize the small intestinal microbiota as a key focus to further advance our understanding of disease pathogenesis and to inform the development of targeted therapeutic strategies.

## Figures and Tables

**Table 1:** Main characteristics and results of principal studies reporting the role of the small intestine in PD.

		Study Groups	Sample Size	Microbiota Sampling Method	Main Results
Preclinical study	Munoz-Pinto et al. [27]	Untreated mice vs. HC ^1^ mice vs. PD ^2^ mice	115 mice (46 untreated; 23 HC ^1^ mice; 46 PD ^2^ mice)	Human fecal material, terminal ileum mucosa biopsies, mouse fecal pellets, and terminal ileum mucosa-associated material	PD dysbiosis may activate a toxic gut-to-brain pathway. Fecal transplants from PD patients into mice can induce immune, functional, inflammatory, and pathological alterations.
Clinical studies	Fasano et al. [26]	PD ^2^ patients vs. HCs ^1^	33 PD ^2^ patients and 30 HCs ^1^	The LBT ^3^ and GBT ^4^ were used to assess the presence of SIBO	SIBO is associated with more severe motor fluctuations
	de Waal et al. [28]	PD ^2^ patients vs. HC ^1^	9 PD patients (6 males, 3 females) and 9 (4 males, 5 females)	Duodenal fluid collected via nasoduodenal tube	Duodenal fluid analysis in PD patients revealed dysbiosis, altered microbial metabolites, and increased α-synuclein accumulation, supporting the role of the small intestine in disease progression.
	Shi et al. [29]	PD group vs. control group	19 patients and 22 controls	Duodenal mucosal biopsies	This study revealed differences in OSyn distribution within the sigmoid mucosa between PD patients and healthy controls; significant changes in the microbiome composition in the gut mucosa of PD patients suggested the potential diagnostic relevance of OSyn/αSyn levels in the sigmoid mucosa for PD.
	trial NCT06003608	PD^2^ patients vs. HC^1^	Total Participants: 100	SIMBA capsule	This clinical trial is ongoing. If this sampling method is effective, it will enable minimally invasive sampling of the microbiome and metabolome from regions of the small intestine that are difficult to reach using conventional approaches.

^1^ Healthy control, ^2^ Parkinson’s disease, ^3^ lactulose breath test, ^4^ glucose breath test.

## Data Availability

Not applicable.
